# Sociodemographic determinants of life satisfaction among grandparent caregivers

**DOI:** 10.3389/fpubh.2023.1044442

**Published:** 2023-01-26

**Authors:** Yi Xu, Ling Zhang, Shuyuan Mao, Si Zhang, Shuzhen Peng, Qi Zhang, Wenwen Wu, Xiaodong Tan

**Affiliations:** ^1^School of Public Health, Wuhan University, Wuhan, Hubei, China; ^2^The Third Affiliated Hospital of Zhengzhou University, Zhengzhou, Henan, China; ^3^Central China Normal University, Wuhan, China; ^4^Huangpi District People's Hospital, Wuhan, China; ^5^Wuhan Health Medical Cosmetic Hospital, Wuhan, China; ^6^School of Public Health, Hubei University of Medicine, Shiyan, China

**Keywords:** personal satisfaction, quality of life, sleep quality (SQ), grandparent caregiver, structural equation modeling

## Abstract

**Introduction:**

It has become a common contemporary phenomenon for grandparents to provide care for young children in their family in both urban and rural areas. This study attempted to investigate psychological wellbeing and quality of life among grandparents involved in childcare in China, and to explore the relationships between sociodemographic characteristics, quality of life, and life satisfaction in this group.

**Methods:**

Using stratified random cluster sampling, we conducted a survey of grandparent caregivers in Wuhan in November and December of 2020 (*N* = 1,640). Descriptive statistics to univariate analysis, multiple linear regression, and structural equation modeling were carried out.

**Results:**

Across all respondents, mean scores on life satisfaction, sleep quality, and quality of life were 14.05 ± 3.50, 5.02 ± 3.37, and 74.51 ± 16.88, respectively. Marital status, income, chronic diseases, family relationships, and physical exercise were found to be associated with life satisfaction. The results of structural equation modeling indicated that quality of life, sleep quality, and sociodemographic characteristics may exert direct and indirect effects on life satisfaction. Mediating effects accounted for 30.0% of the total effects.

**Conclusion:**

Overall, grandparent caregivers have poor life satisfaction, quality of life, and sleep quality. A higher household income, better relationships with family members, healthy lifestyle habits, and high-quality sleep may effectively help to improve life satisfaction among grandparent caregivers.

## 1. Introduction

Life satisfaction, as a subjective factor, is an important component of an individual's subjective experience of wellbeing (1); the term refers to the subjective way in which people perceive and respond to life events. Life satisfaction is a cognitive component of subjective wellbeing and can be defined as an individual's appraisal of the quality of their own life, including the perception that they are progressing toward their goals in life (2). In the literature, life satisfaction has been found to be associated with quality of life, which consists of four dimensions: physical health; psychological health; social functioning and social inclusion; and material circumstances (3).

International studies have highlighted the influence of income, marital status, physical environment, and other factors on life satisfaction (4–6), and have examined the mechanisms by which external factors affect subjective perceptions among adults (6–8). Material wealth (9, 10) and health (11) are considered to be the two most important factors influencing subjective life satisfaction among middle-aged and older adults. Older people who are in poor physical condition or whose financial circumstances are poor report lower levels of life satisfaction (10). Good physical health leads to higher levels of life satisfaction among older adults, while studies reported weaker associations between social support and life satisfaction than might be expected (12). However, a recent study in other populations has demonstrated that social support may affect individuals' physical and mental health (13). Additionally, in a study conducted in Russia, respondents' life satisfaction was found to be influenced by the circumstances of the people surrounding them. If the life satisfaction of their peers was worse than their own, the respondents reported higher levels of life satisfaction (14).

Researchers began to pay increasing amounts of attention to indices of life satisfaction and happiness in China from the 1980's. Most researches has focused primarily on defining the concept of life satisfaction, identifying the factors that influence it, and designing measurement tools for life satisfaction. Studies have shown that the external living environment of urban residents has a significant impact on life satisfaction: for example, individuals report positive emotions, such as optimism, joy, and contentment, if they live in a safe urban area with good access to amenities such as parks and gardens. Major life events, such as divorce or unemployment (15, 16), external living environment, income, views on social equity and justice (6), psychological resilience (13, 17), age (18, 19), community location (20), and social support from family, friends, or other social activities (13, 21) all are factors that have been reported to be associated with life satisfaction and happiness.

Grandparental caregiving has become increasingly common alongside increases in longevity and reductions in fertility (22). According to the Survey of Health, Aging, and Retirement in Europe, over 50% of families make use of grandparental childcare, with the figures for individual countries ranging from 37 to 59% (23). Similarly, approximately 46% of grandparents living in rural China migrate to major cities to provide care for their grandchildren (24). At present, in both urban and rural areas, it has become a widespread phenomenon that grandparents provide care for their grandchildren. Providing grandparental childcare may affect grandparents' quality of life and life satisfaction as they grow older. On the one hand, taking care of children is not only physically challenging, but also causes psychological distress and other negative feelings for grandparents who are required to take on childcare duties, affecting their interactions and relationships with their grandchildren and creating pressure, tension, and distress. On the other hand, taking care of grandchildren also has a range of positive effects, such as improvements in lifestyle, eating habits, physical activity, and social interaction. In addition, providing childcare can enrich the lives of grandparent caregivers by enabling them to maintain a close relationship with their family members. Despite these benefits, the prevalence of grandparental childcare has resulted in increasing concern for the life satisfaction of the grandparents providing this.

**Figure 1:**
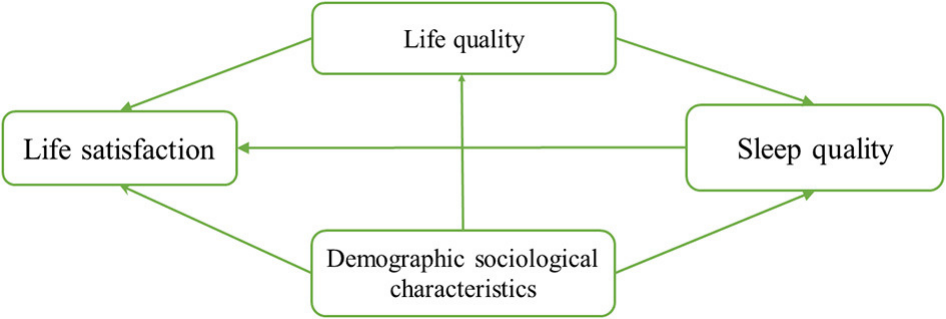
Research framework.

Given the to differences in grandparents participate in family childcare, it is difficult to predict or speculate on their self-perceived quality of life and life satisfaction. Given that previous studies have obtained various outcomes and drawn various conclusions, this study focused on self-perceived levels of life satisfaction and related social determinants of health and wellbeing, such as sleep quality, among grandparent caregivers. The hypothesis was that sleep quality plays a mediating role on the effect of quality of life satisfaction among grandparent caregivers and plays a mediating role in the relationship between quality of life and sociodemographic characteristics on their life satisfaction. Figure 1 shows the conceptual framework employed in this study. The study had two purposes: the first was to assess the life satisfaction of Chinese grandparent caregivers; the second was to investigate the relationships between life satisfaction, quality of life, sleep quality, and sociodemographic characteristics using structural equation modeling (SEM).

## 2. Sampling and methodology

### 2.1. Participants and sampling

This study was conducted using stratified random cluster sampling. During November and December of 2020, participants were recruited from a total of 60 kindergartens and elementary schools in four districts in Wuhan (Wuchang, Hongshan, Donghu High-tech Zone, and Hanyang).

With the help of teachers at each of the kindergartens and elementary schools, grandparents who were taking care of their grandchild were identified. The inclusion criteria for participation in this survey included confirmation that the grandparent had been performing childcare duties for more than 6 months, and that they understood the purpose of the survey and agreed to participate voluntarily. Potential participants who had speech disorders or difficulties with cognitive functioning were excluded. A total of 1,657 questionnaires were distributed as part of this study, and 1,640 valid sets of responses were collected, for a response rate of 98.97%. This study was approved by the research ethics committee of Wuhan University (Approval No. 2020YF0064).

After the survey was complete, the researchers constructed a database using Epidata 3.1, with double entry to ensure the quality of data input. After database cleaning and processing, including the elimination of questionnaires with missing answers or contradictory information, a total of 1,481 cases remained (90.30% of the total received). These correctly completed questionnaires were entered into the analysis.

### 2.2. Measurement instruments

This study used self-administered questionnaires to investigate the psychological wellbeing and quality of life of the grandparents who were included in the sample. The questionnaire consisted of five sections.

In Section 1, sociodemographic information was collected, including gender, age, place of residence, educational status, marital status, employment, monthly household income, the number of children in the household, chronic diseases, the age of the youngest grandchild receiving care from grandparents, employment status of the children's mother, and hired a assistant with childcare.

In Section 2, information was collected on the behaviors and lifestyles of family members, such as smoking, drinking, physical exercise, dietary patterns, and tea drinking.

In Section 3, life satisfaction was evaluated using the Life Satisfaction Index A (LSIA) (25), which consists of 20 items. Possible scores range from 0 to 20. Higher scores indicating higher perceived life satisfaction. This scale is often used to evaluate the life satisfaction of older adults, and has been reported to have good reliability and validity (26, 27).

In Section 4, sleep quality was evaluated using the Pittsburgh Sleep Quality Index (PSQI) (28), which consists of 18 items. The 18 items are categorized into 7 dimensions: (a) subjective sleep quality, (b) sleep latency, (c) sleep duration, (d) habitual sleep efficiency, (e) sleep disturbance, (f) use of sleep medication, and (g) daytime dysfunction; each of these subscales is scored on a scale of 0-3. The total score across each of the 7 dimensions is the total PSQI score, which can range from 0 to 21. The higher the score, the worse the respondent's sleep quality; an overall score above 5 is considered to represent poor sleep quality. Studies have shown that the PSQI has a high degree of reliability and validity in multiple contexts (28).

Finally, in Section 5, quality of life was evaluated using the 36-Item Short Form Health Survey (SF-36), which was originally developed by RAND Health Care and includes eight dimensions: (a) physical functioning, (b) social functioning, (c) role of physical health, (d) body pain, (e) mental health, (f) role of emotion, (g) vitality, and (h) general health. Scores of each of the 8 dimensions are calculated on the basis of the score for each relevant item. The raw score on each dimension is the sum of the scores for all items on this dimension. The final score for each dimension is obtained by adjusting the raw score as follows: final score = [(actual score - lowest possible score for that dimension)] / [(highest possible score for that dimension - lowest possible score for that dimension)] × 100. The higher the respondent's SF-36 score, the better their quality of life is. Numerous studies have verified the reliability and validity of the Chinese version of the SF-36 (29).

### 2.3. Statistical analysis

SPSS 22.0 and Amos 22.0 were used for statistical analysis in this study. Descriptive statistics, univariate analysis and multiple linear regression analyses were performed. Descriptive statistics are reported to describe the sociodemographic characteristics of respondents. *T*-tests or analyses of variance were used for numerical variables. Univariate analysis and multivariate linear regression were used to explore the relationships between sociodemographic characteristics, sleep quality, quality of life, and life satisfaction. Subsequently, structural equation models were constructed to identify mediating effects on associations of sociodemographic characteristics, sleep quality, and quality of life with life satisfaction. We evaluated the adjusted model by the basis of the chi-squared goodness-of-fit test (χ^2^/df ≤ 3.00 indicates a good model fit), the root mean square error of approximation (RMSEA; values ≤ 0.08 indicate a very good fit), the goodness of fit index (GFI), the Tucker–Lewis index (TLI), the comparative fit index (CFI), and the incremental fit index (IFI; values ≥0.90 indicate a very good fit).

## 3. Results

### 3.1. Sociodemographic characteristics

A total of 1,481 participants were included in this study, 26.47% of whom were men. The average age across all participants was 62.11 ± 5.98 years. In terms of education, approximately 28.63% of respondents had completed high school or technical secondary school, while 20.46% had an elementary or lower level of education. More than half of respondents (56.18%) were retired, while 16.47% were employed. More than a quarter of respondents (27.35%) were unemployed.

Only 6.62% of respondents provided care to a grandchild more than 6 years old; more than half (52.67%) provided care to a grandchild between 3 and 6 years old, while 13.37% provided care to a grandchild between 1 and 3 years old, and 27.34% provided care to a grandchild under one year old. Most of the respondents (60.16%) reported having good relationships with other members of their family. The vast majority of respondents (95.07%) indicated that they did not employ household workers for assistance with childcare or housework.

Regarding health behaviors, only 10.13% of respondents were smokers, and only 12.15% of respondents reported drinking alcohol. Almost half of participants (48.75%) reported engaging in regular physical exercise. Approximately 80% of respondents reported eating a diet consisting of a balance between meat and vegetables, while 16.75% were vegetarians. Detailed information is presented in Table 1.

**Table 1:** Univariate analysis of factors influencing life satisfaction among grandparent caregivers.

**Variable**	**Category**	***n* (%)**	**Life satisfaction score (M ±SD)**	**t/F**	***P***
Gender	Male	392 (26.47)	13.89 ± 3.45	−1.027	0.305
	Female	1,089 (73.53)	14.10 ± 3.52		
Age (years)	< 60	521 (35.17)	14.21 ± 3.48	1.44	0.237
	60–70	782 (52.80)	14.01 ± 3.55		
	≥70	178 (12.02)	13.71 ± 3.30		
Residence	Urban	1290 (87.10)	14.19 ± 3.45	4.094	< 0.001
	Rural	191 (12.90)	13.08 ± 3.71		
Education	Primary or below	303 (20.46)	13.26 ± 3.57	8.576	< 0.001
	Junior high school	372 (25.12)	13.88 ± 3.53		
	High school or technical secondary school	424 (28.63)	14.42 ± 3.45		
	Junior college or above	382 (25.79)	14.41 ± 3.35		
Marital status	Married	1,268 (85.62)	14.19 ± 3.45	4.016	< 0.001
	Other	213 (14.38)	13.16 ± 3.68		
Employment status	Employed	244 (16.47)	14.11 ± 3.26	3.984	0.019
	Retired	832 (56.18)	14.23 ± 3.57		
	Unemployed	405 (27.35)	13.63 ± 3.46		
Monthly household income (yuan)	≤ 1,000	153 (10.33)	12.88 ± 3.56	16.348	< 0.001
	1,001–3,000	405 (27.35)	13.65 ± 3.54		
	>3,000	923 (62.32)	14.41 ± 3.41		
Number of children	1	750 (50.64)	14.19 ± 3.56	3.227	0.040
	2	528 (35.65)	14.06 ± 3.33		
	≥3	203 (13.71)	13.49 ± 3.64		
BMI (kg/m^2^)	< 18.5	79 (5.33)	13.46 ± 3.63	4.085	0.007
	18.5–23.9	880 (59.42)	14.25 ± 3.48		
	24–26.9	395 (26.67)	13.98 ± 3.36		
	≥27	127 (8.58)	13.23 ± 3.82		
Number of chronic diseases	0	31 (2.09)	14.87 ± 3.81	18.71	< 0.001
	1	954 (64.42)	14.48 ± 3.28		
	2	263 (17.76)	13.54 ± 3.69		
	≥3	233 (15.73)	12.74 ± 3.72		
Age of youngest grandchild (months)	≤ 12	405 (27.34)	13.73 ± 3.43	2.271	0.079
	13–36	198 (13.37)	13.99 ± 3.70		
	37–72	780 (52.67)	14.15 ± 3.52		
	>72	98 (6.62)	14.64 ± 3.10		
Children's mother at home full-time?	Yes	311 (21.00)	14.06 ± 3.48	0.068	0.946
	No	1,170 (79.00)	14.04 ± 3.51		
Who the respondent lives with	Spouse	332 (22.42)	13.99 ± 3.55	1.717	0.162
	Spouse and child	400 (27.01)	14.35 ± 3.21		.
	Child	429 (28.97)	13.80 ± 3.67		
	Other	320 (21.61)	14.04 ± 3.56		
Relationships with family members	Very good	891 (60.16)	14.83 ± 3.16	95.66	< 0.001
	Good	447 (30.18)	13.67 ± 3.34		
	Worse	143 (9.66)	10.94 ± 3.91		
Employ a nanny or part-time household worker?	Yes	73 (4.93)	13.97 ± 3.52	−0.184	0.854
	No	1,408 (95.07)	14.05 ± 3.50		
Smoking	Do not smoke	1,220 (82.37)	14.10 ± 3.53	0.85	0.428
	Smoker	150 (10.13)	13.75 ± 3.58		
	Have given up smoking	111 (7.50)	13.85 ± 3.03		
Drinking	Do not drink	1,231 (83.12)	14.10 ± 3.50	1.756	0.173
	Drinker	180 (12.15)	13.95 ± 3.57		
	Have given up drinking	70 (4.73)	13.31 ± 3.26		
Exercise every day?	Yes	722 (48.75)	14.83 ± 3.17	8.662	< 0.001
	No	759 (51.25)	13.30 ± 3.63		
Dietary preferences	Meat-based	52 (3.51)	13.54 ± 2.96	4.055	0.018
	Vegetarian	248 (16.75)	13.53 ± 3.81		
	Balanced diet	1,181 (79.74)	14.18 ± 3.44		
Drink tea every day?	Yes	449 (30.32)	14.40 ± 3.40	2.548	0.011
	No	1,032 (69.68)	13.89 ± 3.56		

### 3.2. Sleep quality

The average PSQI score was 5.02 ± 3.37. Considering the threshold score of 5, 36.26% of respondents reported experiencing sleep problems. Across all participants, 14.11% of respondents reported “poor” subjective sleep quality, and 2.91% of respondents reported that their subjective sleep quality was “pretty poor.” Detailed information is presented in Table 2.

**Table 2:** Sleep quality among grandparent caregivers.

**Variable**	***N***	**%**
**Sleep quality**		
≤ 5	944	63.74
>5	537	36.26
**Subjective sleep quality**		
Pretty good	455	30.72
Good	774	52.26
Poor	209	14.11
Pretty poor	43	2.91

In addition, 24.92% of participants reported taking 31–60 min to fall asleep, while 5.54% needed more than 60 min. Over half of respondents (50.30%) reported sleeping more than 7 h per night, and 27.14% indicated that they slept <6 h per night. Furthermore, approximately 65.43% of the respondents reported a sleep efficiency higher than 85%, while efficiency was below 65% for 7.29% of respondents. The proportion of respondents with a severe sleep disorder (with an overall score of 2 or 3) was 20.32%, while the proportion of respondents who had sleep medication during the past month was 3.65%. Nearly half of respondents (44.90%) reported experiencing daytime dysfunction due to sleep disturbances.

### 3.3. Quality of life

The average SF-36 score among all respondents was 74.51 ± 16.88, where higher scores indicate a higher quality of life. Across all participants, the average score of dimensions relating to physical health was 75.37 ± 18.44, and the average score of dimensions relating to psychological health was 73.65 ± 17.80. Among all dimensions of the SF-36, the highest-scoring was body pain, with an average score of 80.92 ± 17.14. Detailed information is presented in Table 3.

**Table 3:** Quality of life among grandparent caregivers.

**Variable**	**M**	**SD**
**Overall quality of life score**	74.51	16.88
**Physical component overall score**	75.37	18.44
Physical functioning	79.66	18.39
Body pain	80.92	17.14
Role of physical health	76.11	37.64
General health	64.79	19.75
**Mental component overall score**	73.65	17.8
Vitality	68.7	15.63
Social functioning	78.96	20.27
Role of emotion	77.85	37.07
Mental health	69.08	16.62

### 3.4. Associations between sociodemographic characteristics and life satisfaction

The average score of life satisfaction among all respondents was 14.05 ± 3.50. Univariate analysis indicated several variables were associated with life satisfaction. The relevant factors included (1) residence, (2) education, (3) marital status, (4) employment, (5) family income, (6) number of children in the household, (7) BMI, (8) chronic illnesses, (9) family relationships, (10) physical activities, (11) diet, and (12) tea drinking. These variables each had a significant impact on subjective life satisfaction among grandparent caregivers. Specifically, a higher level of education, a higher monthly family income, and good family relationships were beneficial for life satisfaction among grandparent caregivers. The full results of this analysis are presented in Table 1.

### 3.5. Multiple linear regression analyses of the influence of sociodemographic characteristics on life satisfaction

Univariate analysis indicated that there were multiple factors affecting life satisfaction among grandparent caregivers. In order to further explore the relationships between these factors and life satisfaction, a multiple linear regression model was constructed. After adjustment for related variables, respondents who were not married or in a committed relationship were found to report lower levels of life satisfaction in comparison to married respondents. Respondents with a monthly household income of more than 3,000 yuan had higher life satisfaction scores than those with a lower monthly income. Respondents with one or more chronic diseases reported lower levels of life satisfaction in comparison to those with no chronic diseases. Respondents with negative relationships with their family members had lower levels of life satisfaction in comparison to those with positive relationships. Furthermore, life satisfaction was lower among participants who did not engage in regular exercise than among those who routinely engaged in physical exercise. The details of these results are presented in Table 4.

**Table 4:** Multivariate analysis of factors influencing life satisfaction among grandparent caregivers.

**Variable**		**β**	**Sb**	**t**	***P***
Constant		15.593	0.731	21.34	< 0.001
**Residence (Reference: Urban)**					
	Rural	−0.382	0.282	−1.35	0.176
**Education (Reference: Primary or below)**					
	Junior high school	0.234	0.260	0.90	0.368
	High school or technical secondary school	0.433	0.278	1.56	0.120
	Junior college or above	0.205	0.299	0.69	0.493
**Marital status (Reference: Married)**					
	Other	−0.823	0.239	−3.44	0.001
**Number of children (Reference: 1)**					
	2	0.213	0.200	1.07	0.286
	≥3	0.060	0.289	0.21	0.836
**Employment status (Reference: Employed)**					
	Retired	0.312	0.248	1.26	0.209
	Unemployed	0.530	0.294	1.80	0.072
**Monthly household income (Yuan; reference:** = ≤ **1,000)**					
	1,000–3,000	0.507	0.329	1.54	0.123
	>3,000	0.857	0.327	2.62	0.009
**BMI (kg/m** ^2^ **; reference: 18.5–23.9)**					
	< 18.5	−0.454	0.374	−1.21	0.225
	24–26.9	−0.119	0.193	−0.61	0.539
	≥27	−0.558	0.305	−1.82	0.069
**Number of chronic diseases (Reference: 0)**					
	1	−0.753	0.589	−1.28	0.201
	2	−1.414	0.612	−2.31	0.021
	≥ 3	−2.050	0.615	−3.33	0.001
**Relationships with family members (Reference: Very good)**					
	Good	−1.132	0.186	−6.09	< 0.001
	Neutral or worse	−3.297	0.293	−11.25	< 0.001
**Exercise every day? (Reference: Yes)**					
	No	−1.152	0.169	−6.82	< 0.001
**Dietary preferences (Reference: Balance of meat and vegetables)**					
	Meat-based	−0.633	0.454	−1.40	0.163
	Vegetarian	−0.363	0.225	−1.62	0.106
**Drink tea every day? (Reference: Yes)**					
	No	−0.282	0.184	−1.53	0.126

F = 15.56, P < 0.001.

**Figure 2:**
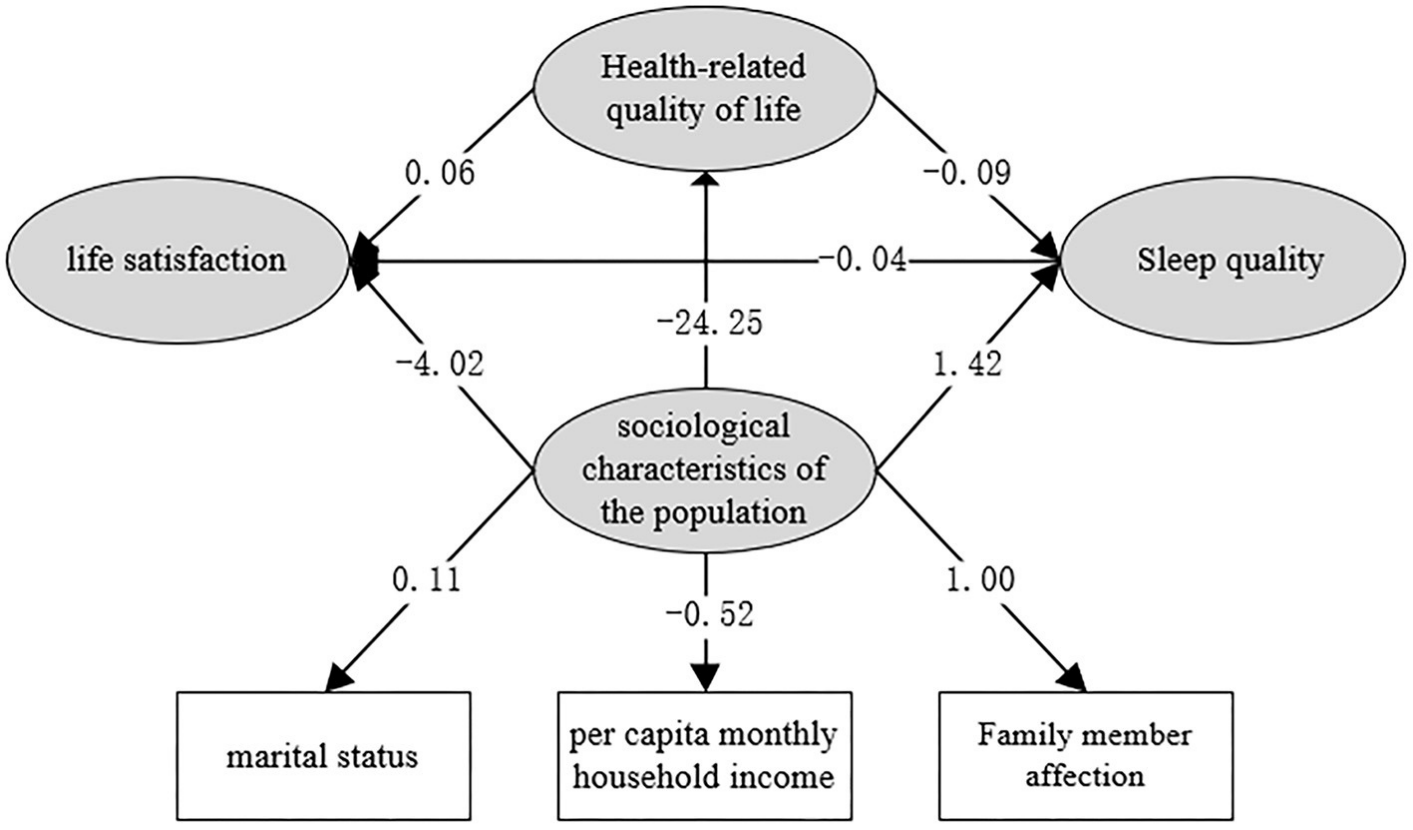
Structural equation model path coefficients (unnormalized).

Sociodemographic, sociological, and lifestyle factors identified as relevant in the multivariate analysis, along with quality of life and sleep quality, were entered into the structural equation modeling analysis. Structural equation models were constructed to identify the factors influencing life satisfaction using the Maximum Likelihood method. Path relationships between each of the variables were calculated, and indicators of model fit were subsequently computed. Differences between models were considered to be statistically significant at P < 0.05. The mediation model represented a good fit to the data: χ^2^/df =2.28; RMSEA = 0.0029; CFI = 0.997 (see Table 5). The direct, indirect, and total effects of quality of life on life satisfaction were 0.31, 0.02, and 0.33, respectively. The total effect of sleep quality score on life satisfaction was −0.04. Finally, the direct, indirect, and total effects of sociodemographic characteristics on life satisfaction were −0.42, −0.17, −0.59, and respectively. Full details of the direct and indirect effects and the mediation model are presented in Table 6 and Figure 2.

**Table 5:** The fitness of SEM.

**Criterion**	**Criteria**	**Result**	**Judge**
χ^2^/df	< 5	2.28	Accepted
NFI	>0.9	0.989	Accepted
RMSEA	< 0.05	0.029	Accepted
TLI	>0.9	0.985	Accepted
GFI	>0.9	0.997	Accepted
CFI	>0.9	0.994	Accepted

NFI, Normed Fit Index; RMSEA, root mean square error of approximation; TLI, Tucker–Lewis index; GFI, Goodness of fit index; CFI, comparative fit index.

**Table 6:** Direct and indirect effects of quality of life and sleep quality on life satisfaction.

	**Total effect**	**Direct effect**	**Indirect effect**
Quality of life	0.33^*^	0.31^*^	0.02^*^
Sociological characteristics	−0.59^*^	−0.42^*^	−0.17^*^
Sleep quality	−0.04^*^	−0.04^*^	-

^*^p < 0.001.

## 4. Discussion

Based on a survey of grandparent caregivers in Wuhan, this study attempted to investigate the psychological wellbeing and quality of life of grandparents involved in childcare, and to explore the relationships between sociodemographic characteristics, quality of life, and life satisfaction in this population. Various sociodemographic characteristics were found to be associated with life satisfaction, and mediating effects of quality of life and sleep quality were also found to be in operation in this relationship. A chain effects of sociodemographic characteristics-quality of life- sleep quality- life satisfaction was also observed. Physical exercise, quality of life, and sleep quality were significant predictors of life satisfaction among grandparent caregivers.

Higher life satisfaction scores were observed among respondents who were married or in a committed relationship, which is consistent with results reported by Lee et al. (8). Presumably, people who live alone or are unmarried, divorced, or widowed tend to lack care from their peers as well as interaction and connection with others, and they are prone to feeling lost or lonely. The relationship between marital status and health (30, 31), including physical and mental health (4, 32, 33), has also been confirmed by many studies.

Consistent with the findings of Smith et al. (34), higher household income was found to be associated with higher life satisfaction. Increases in income are always accompanied by improvements in living conditions and opportunities relating to residence, diet, lifestyle, and social activities. Members of low-income families are at a higher risk of experiencing negative life events, such as unemployment, divorce, separation, and poor family relationships. These negative life experiences and challenges place older adults at a higher risk of experiencing emotional disorders, such as sadness, depression, hopelessness, nervousness, helplessness, or lacking of purpose. Previous studies have reported that people with a low income or who are unemployed have lower life satisfaction (5).

In line with the findings of Garrison's (1998) study (35), we observed lower levels of life satisfaction among respondents with one or more chronic medical conditions. According to previous studies, poor health is detrimental to mental health and happiness, affecting an individual's daily life and reducing their life satisfaction (4). Consistent with previous studies (31), our findings indicated a positive correlation between life satisfaction and engagement in physical exercise. The positive effects of physical exercise on life satisfaction can be explained by the fact that regular exercise was also associated with a lower risk of chronic disease, good sleep quality, and higher quality of life. High-quality sleep has a positive effect on physical and mental health, as the body needs high-quality sleep in order to be prepared for the next day. In order to achieve the grand goal of “healthy aging” proposed by the WHO in 1993, scholars all over the world have investigated the various factors that can affect quality of life among people of all demographic groups, not only older adults. Sleep quality has been found to be a crucial contributing factor in quality of life. Existing studies have also shown that factors such as age (36), sex (37, 38), sleep habits (39), physical illnesses and chronic conditions (40), mental illness (41), environmental factors (42), psychosocial factors (43), and treatment for insomnia (44) affect sleep quality among older adults.

According to previous studies, close social relationships may exert a powerful influence on physical health and wellbeing in later adulthood (45). In our study, respondents who had negative relationships with their family members reported lower levels of life satisfaction than those whose family relationships were positive. This can probably be attributed to the psychological strain of living in a household where there is a lack of positive communication and interaction. There is a consensus across both Eastern and Western studies that family relationships affect life satisfaction among grandparents (46). However, a study of cultural differences between China and the United States has suggested that family relationships are closely related to life satisfaction among Chinese older adults, while relationships with friends are more closely associated with life satisfaction among American older adults (47). Given that China's social security system and social pension resources are still relatively weak, family pensions are still the main form of pension in China. Therefore, older adults with harmonious family relationships are prone to achieve higher levels of life satisfaction.

In this study, several sociodemographic characteristics were found to be associated with life satisfaction, as well as quality of life, among grandparent caregivers. Additionally, sociodemographic characteristics and quality of life exerted indirect effects on life satisfaction *via* sleep quality. Therefore, increasing household income, improving family relationships, building healthy lifestyle habits, and improving sleep quality may help to improve levels of life satisfaction among grandparent caregivers.

Several limitations of this study should be noted. First, although the subjective scales used in this study have been demonstrated to be reliable and valid in multiple studies, our results may inevitably deviate quantifiably from objective reality. In addition, the study was limited to a specific geographical area, and the results therefore cannot be extrapolated to other regions without certain methodological innovations. Finally, this study was cross-sectional in nature and no longitudinal data were collected, which limits the extent to which causal relationships between variables can be explored. Cohort studies could be carried out in future to determine whether the relationships identified in this study are in fact causal and not simply incidental.

## 5. Conclusion

Various sociodemographic characteristics may affect life satisfaction, with quality of life and sleep quality playing mediating roles in this relationship. A higher household income, better relationships with family members, healthy lifestyle habits, and high-quality sleep may effectively help to improve life satisfaction among grandparent caregivers.

## Data availability statement

The raw data supporting the conclusions of this article will be made available by the corresponding authors upon reasonable request.

## Author contributions

XDT: conceptualization. YX, LZ, SYM, and SZ: methodology and formal analysis. XDT, YX, LZ, SYM, and SZ: validation. YX, LZ, SZ, SZP, QZ, and WWW: investigation. XDT, YX, and WWW: resources. YX, LZ, SZ, and WWW: data curation. YX, LZ, and SYM: writing–original draft preparation. XDT and WWW: writing–review and editing and supervision. LZ and SYM: visualization. All authors have read and agreed to the published version of the manuscript.
